# Moral Injury Among Physicians Caring for Immigrant Patients Amid Anti-Immigrant Policies

**DOI:** 10.1001/jamainternmed.2026.2899

**Published:** 2026-07-20

**Authors:** Marlene Martín, Lupita Ambriz, Mario Gonzalez Ramirez, Rahael Borchers, Lucy B. Schulson, Lilia Cervantes, Alicia Fernández

**Affiliations:** 1 Department of Medicine, University of California, San Francisco; 2 Latinx Center of Excellence, University of California, San Francisco; 3 San Francisco General Hospital, San Francisco, California; 4 Section of General Internal Medicine, Boston Medical Center, Boston, Massachusetts; 5 Chobanian and Avedisian School of Medicine, Boston University, Boston, Massachusetts; 6 Department of Medicine, University of Colorado, Aurora

## Abstract

**Importance:**

Beginning in January 2025, the sociopolitical context in which immigrant patients seek and receive health care has changed significantly.

**Objective:**

To explore the perspectives of primary care physicians regarding their own well-being and the health and well-being of their immigrant patients after anti-immigrant policy changes in January 2025.

**Design, Setting, and Participants:**

This qualitative study used virtual semistructured interviews with primary care physicians across the US (recruited using purposive and snowball sampling) who were providing health care to immigrant patients. The study was conducted between July and September 2025. The audio from the physician interviews was recorded, transcribed, and analyzed using thematic analysis.

**Main Outcomes and Measures:**

Themes and subthemes derived from interviews.

**Results:**

There were 38 participants interviewed (mean age, 40.3 [SD, 10.8] years; 27 women [71.1%]; by specialty: 24 [63.2%] internal medicine, 13 family medicine [34.2%], and 1 internal medicine–pediatrics [2.6%]). The identified themes and subthemes were (1) drivers of physician moral injury (ethical and professional values conflict, compounded burden on immigrant identities, workforce strain, powerlessness, navigating dynamic policies, altering clinical decision-making); (2) patient vulnerability and health consequences (complex decision-making within mixed-status families, ripple effects on US citizens and broader communities, pervasive fear and hesitancy of accessing care, physical and mental health deterioration); (3) contrasting responses from health care systems (restricting health care, institutional silence and suppression of immigration-specific support, adapting through flexibility in care delivery, guiding through proactive communication and partnerships); and (4) physician empowerment (engaging in multilevel advocacy, leveraging trust, role modeling for trainees and peers, protecting patient privacy).

**Conclusions and Relevance:**

In this qualitative study, physicians experienced moral injury stemming from anti-immigrant policies starting in January 2025, which was compounded for those with immigrant identities and because of health care systems’ varying responses to these policies. Physicians perceived that changes in immigration policy had adversely affected immigrant and US citizens. Despite these challenges, physicians and health care systems have adapted to support patients and staff, which has mitigated moral injury.

## Key Points

**Question:**

What are the experiences of 38 primary care physicians caring for immigrant patients since January 2025?

**Findings:**

In this qualitative study, participants reported moral injury stemming from anti-immigrant policies, which was exacerbated by the adverse effects of the policies on the health of immigrant and US citizen patients, and by health care systems that limited immigrant patient support or remained silent. Moral injury was mitigated by supportive health care system responses and physician advocacy.

**Meaning:**

Anti-immigrant policies have adversely affected the well-being of physicians, immigrants, and US citizens; supportive health care system responses and physician advocacy may help mitigate anti-immigrant policy harms and reduce moral injury.

## Teaser

This qualitative study explores the perspectives of primary care physicians regarding their own well-being and the health and well-being of their immigrant patients after anti-immigrant policy changes in January 2025.

## Introduction

Increased US immigration enforcement and anti-immigrant policies have dramatically altered the sociopolitical context in which immigrants—including authorized (73% of the immigrant population) and undocumented individuals (all henceforth referred to as immigrants)—interact with health care systems.^1,2^ In January 2025, a US executive order broadened immigration law enforcement.^3^ Under the protected areas policy, locations including hospitals, schools, and churches, were protected from US Immigration and Customs Enforcement (ICE) but this policy was rescinded in January 2025.^4^ Congress also allocated $170 billion to the US Department of Homeland Security (DHS) to increase deportations, and the Centers for Medicare & Medicaid Services agreed to share data with the DHS to support immigration enforcement.^5,6,7,8^

Highly publicized ICE activity and arrests, including in or near hospitals, have intensified fear among immigrants across large US cities.^9,10,11^ These actions have created a substantial *chilling effect*, in which heightened fear leads to reduced health care use among undocumented individuals and mixed-status families—a phenomenon documented during prior periods of increased immigration enforcement.^12,13,14,15,16,17,18,19,20,21,22,23^ In turn, detrimental effects on access to care can generate moral injury, which is the distress clinicians may experience when providing care that conflicts with deeply held ethical values.^24,25,26^

Understanding physicians’ experiences caring for immigrant patients is essential to exploring if anti-immigrant policies generate moral injury. To examine the effects of anti-immigrant policy since January 2025, we conducted a qualitative study exploring the perspectives of primary care physicians regarding their own well-being and the well-being of their immigrant patients in the current sociopolitical climate.

## Methods

### Study Design, Setting, and Participants

We conducted semistructured 1-on-1 interviews between July and September 2025. Participants received $50 remuneration and provided verbal consent and permission to have parts of their interviews published. Eligible participants included third-year residents and attending physicians trained in family medicine, internal medicine, or internal medicine–pediatrics who reported providing health care to adult immigrant patients in the US. Physicians in Texas and Florida were excluded because these states required health systems to document patient immigration status before 2025, which may have had a chilling effect.^27,28,29^

We recruited physicians using purposive and snowball sampling via email, including outreach to division chiefs, individual physicians, and an immigrant health group listserv hosted by the Society of General Internal Medicine. This study followed the Consolidated Criteria for Reporting Qualitative Research (COREQ) reporting guideline.^30^ An institutional review board at the University of California, San Francisco, approved the study as exempt.

### Interview Guide

The interview guide was informed by literature review^17,18,19,20,21,24^ and author group expertise and iteratively refined. The questions explored the perspectives of physicians on the health and well-being of their immigrant patients, physician well-being, strategies physicians and their clinical settings have used to provide care for immigrant patients, and recommendations to improve immigrant care. We asked physicians about the experiences of their immigrant patients to understand how these contextual observations shaped physician well-being rather than as direct representations of the lived experiences of patients. The participant interview guide appears in Supplement 1.

### Data Collection

Participants completed a brief survey capturing demographic and clinical practice characteristics. Four trained qualitative interviewers (M.M., L.A., M.G.R., and R.B.) conducted the interviews via Zoom version 7.0.2. All interviewers identify as children of immigrants and include a Latina physician-researcher, a South Asian biracial physician-researcher, and 2 Latino research analysts. Interviewers introduced their role and interest in the study before commencing interviews. The audio from the interviews was recorded, transcribed verbatim using Zoom’s artificial intelligence assistant, reviewed for accuracy, deidentified, and conducted until thematic saturation.^31,32^

### Data Analysis

Coding and analysis occurred between August and December 2025 using thematic analysis.^31^ Three of the authors (M.M., M.G.R., and L.B.S.) independently line-by-line inductively coded 5 interviews using Dedoose version 10.0.35 (Sociocultural Research Consultants LLC) to develop an initial codebook. The team compared coding and resolved discrepancies through discussion. They coded remaining transcripts in groups of 4, identifying emergent codes and resolving discrepancies during weekly meetings. Two of the authors (M.M. and L.A.) then grouped the codes into candidate themes and subthemes, which were iteratively reviewed with the full team. The broader analytic team included 2 additional Latina immigrant physician-researchers (L.C. and A.F.) and reflexivity was supported through ongoing discussions and documentation of analytic decisions. Interpretations were refined through team consensus and member checking.

## Results

There were 73 individuals who expressed interest in participating. Of the 73 individuals, 67 met study criteria and 38 were interviewed. Among the 38 participants, 3 expressed fear of repercussion for participating, 27 (71.1%) were women and the mean age was 40.3 (SD, 10.8) years (Table 1). Of the 38 participants, 32 (84.2%) practiced in urban settings and 28 (73.7%) practiced in academic settings. The mean interview duration was 32.4 (SD, 10.6) minutes. Participants represented 13 states (Table 1), which included cities with increased immigration enforcement (eg, Los Angeles, California; New York, New York; and Boston, Massachusetts). When attached to quotes, participant locations are grouped by region to increase confidentiality.^33^ We identified 4 themes and 18 subthemes (illustrative quotations appear in Table 2). A conceptual model displaying the drivers and mitigators of physician moral injury due to anti-immigrant policies appears in the Figure. The recommendations made by the participants to support immigrant patients appear in Table 3.

**Table 1.** Physician Characteristics

Characteristic	Physicians, No. (%) (N = 38)
Age, mean (SD), y	40.3 (10.8)
Gender	
Man	9 (23.7)
Woman	27 (71.1)
Other gender identity	2 (5.3)
Race and ethnicity^a^	
Asian	12 (31.6)
Black	1 (2.6)
Latino	8 (21.1)
White	20 (52.6)
Other^b^	2 (5.3)
Clinical characteristics	
Attending physician	31 (81.6)
Resident physician	7 (18.4)
Medical director or clinical operations	5 (19.2)
Physician specialty	
Internal medicine	24 (63.2)
Family medicine	13 (34.2)
Internal medicine–pediatrics	1 (2.6)
Patient care, % of time spent^c^	
0-25	9 (23.7)
26-50	9 (23.7)
51-75	4 (10.5)
76-100	16 (42.1)
Year graduated from medical school, median (IQR)	2016 (2010-2019)
Location	
West^d^	20 (52.6)
Northeast^e^	15 (39.5)
South^f^	3 (7.9)
Midwest	0
Clinical practice setting^a^	
Urban	32 (84.2)
Suburban	6 (15.8)
Rural	1 (2.6)
Type of clinical practice^a^	
Academic or affiliated with academic institution	28 (73.7)
Community	18 (47.4)
Safety net	4 (10.5)
Other	1 (2.6)

^a^ The categories are not mutually exclusive.

^b^ Included Middle Eastern and Jewish.

^c^ Direct patient care or supervising teams including residents.

^d^ Includes participants from California, Colorado, Idaho, New Mexico, and Washington.

^e^ Includes participants from Connecticut, Massachusetts, New York, Pennsylvania, and Rhode Island.

^f^ Includes participants from Kentucky, Maryland, and Tennessee.

**Table 2.** Themes and Subthemes With Participant Illustrative Quotes

Themes and subthemes	Participant illustrative quotes
**Theme 1: drivers of physician moral injury**	
Ethical and professional values conflict	“[The policies are a] setup for bad outcomes and in conflict with the philosophy of health care and serving humanity…and what we signed up for and have a responsibility to do,” said participant No. 332 located in the Northeast.
	“I don’t have a good sense of the risks associated with what people must do to navigate the health care system [right now]. The uncertainty is very troubling and takes away a lot of what I feel to be an essential part of my role as somebody’s primary care doctor,” said participant No. 362 located in the South.
Compounded burden on immigrant identities	“We’re not many Latinas here. I’m the only one among 15 providers…that’s a minority, and I feel very lonely,” said participant No. 355 located in the West.
	“I’m very anxious. My mom is completely freaked out and really wants me to get a non-American passport because if she gets deported, she’s like ‘Where am I going to be deported to? I’m from Cuba. You have to give up your citizenship to come here’ and she’s a refugee,” said participant No. 349 located in the Northeast.
	“As a caregiver for an immigrant child I have to be careful about not having a public presence,” said participant No. 347 located in the Northeast.
Workforce strain	“We had 3 medical assistants who were here on TPS, and their TPS was repealed so they had to be terminated, which was horrible,” said participant No. 333 located in the Northeast.
	“It’s taking a ton more of my time in terms of paperwork. Especially with refugees…they don’t have community organizations to do it anymore,” said participant No. 321 located in the Northeast.
Powerlessness	“…this feels very helpless because the things that we want to do for people, oftentimes we can’t, and the things we’re able to do are just the smallest drop in the bucket of the life problems that they have going on that we’re powerless to fix,” said participant No. 322 located in the Northeast.
	“I can burst into tears at almost any moment because the burden and the weight of the grief is enormous. Both the cruelty of what is happening in this country, and with our governmental policies, which are very violent,” said participant No. 318 located in the West.
Navigating dynamic policies	“It [the policy to document citizenship status and only provide federally funded services to citizens] was rolled out as if it’s active right now…[our lawyer was] waiting for the ACLU to file a lawsuit, but until they do that we have no idea what to do with this…,” said participant No. 334 located in the West.
	“DHHS released a notice that any center that receives federal funding will not be allowed to serve patients who are undocumented anymore…our clinic’s administration explained that this is something that we are in process of reviewing legally,” said participant No. 337 located in the Northeast.
Altering clinical decision-making	“Am I not sending this person to the emergency department because they don’t need to go to the emergency department clinically or is it because I’m afraid that they’re increasing their risk of being deported?” said participant No. 324 located in the Northeast.
	“I might be starting a medication that I normally wouldn’t have started as soon…trying to fit more in because [of] concern or uncertainty about what the insurance landscape [is] going to look like in a few months,” said participant No. 345 located in the West.
**Theme 2: patient vulnerability and health consequences**	
Complex decision-making within mixed-status families	“I have one [patient] who has a daughter with special needs, and she was petrified of family separation. Her daughter’s an American citizen.... She decided to go back to her home country with her daughter, and she asked me to prescribe 3 months of medications so she could take those with her, while she figures out a plan of care…,” said participant No. 362 located in the South.
	“Latino parents would…buy their children food and clothing before ever purchasing medicines for themselves. Budgetwise [for those who are now unemployed], health is now at the bottom of a list of things that are not being fulfilled right now,” said participant No. 334 located in the West.
Ripple effects on US citizens and broader communities	“They are threatening people’s safety such that no one should feel okay and safe…whether you are here as a person with documented status or not…we’re seeing the worries and the impacts of that,” said participant No. 356 located in the Northeast.
	“It also is affecting people who are scared that they may be typecast into not having documentation status. People are scared that their naturalization is going to be removed,” said participant No. 324 located in the Northeast.
Pervasive fear and hesitancy of accessing care	“I go into the room and [the patient says] ‘I don’t want to take [my medications] because I don’t want to go to the pharmacy to pick up my [medications] because I’m concerned about deportation…,’” said participant No. 351 located in the West.
	“[She] had a really severe bike accident a week prior…and was having terrible headaches after the injury and never went to the emergency room because she was too afraid,” said participant No. 327 located in the Northeast.
Physical and mental health deterioration	“My resident saw a patient who said that his son had been detained by ICE in [a Northeast state]…this patient had a history of well-controlled diabetes, but due to the stress of this situation, his [hemoglobin] A_1C_ had become elevated to 10,” said participant No. 329 located in the Northeast.
	“I had a suicide attempt the other day of a patient who is in a mixed-status family with a lot of stressors around…. I don’t often see suicide attempts in patients who are in their 50s and 60s, but she also had not been going to the doctor because of fear,” said participant No. 341 located in the West.
**Theme 3: contrasting responses from health care systems**	
Restricting health care	“We’ve been asked to see fewer of our undocumented or uninsured…. I see 10 to 12 patients per half day, and only 2 or 3 of those can be uninsured patients…for the financial well-being of the clinic…even though our uninsured population accounts for half of our patient population,” said participant No. 342 located in the West.
Institutional silence and suppression of immigration-specific support	“[Leadership] doesn’t communicate…. [They] don’t want to face any pushback from [any messaging or activism]. [They] don’t want to lose more funding…. They’re trying to get the best of both worlds because [the hospital] does advertise itself as a safe haven and a safety net hospital,” said participant No. 349 located in the Northeast.
	“How frustrating it has been to be told by our lawyers, who are just trying to protect the organization, that we as physicians can’t speak out,” said participant No. 338 located in the West.
	“…our clinic has not developed any protocol to move patients from the waiting room into the clinical area. No protocol on how to call an overhead announcement to get all of us to come to the lobby to protect the front office staff…,” said participant No. 318 located in the West.
Adapting through flexibility in care delivery	“We’ve been working in our group to think through how we make sure that people know that they can use [telemedicine] and what the barriers are to using [telemedicine],” said participant No. 356 located in the Northeast.
	“We’re working out a workflow [to address no shows] to codify it in our clinic…the medical assistant will call every patient who no shows and offer them a telephone visit,” said participant No. 319 located in the West.
Guiding through proactive communication and partnerships	“Our hospital set up…[an] immigrant health task force that’s meant to bring together stakeholders from across different parts of the hospital, like folks from government advocacy, nursing, marketing, and legal,” said participant No. 356 located in the Northeast.
	“We’ve partnered with organizations to provide information for our patients to be aware of their rights…we have clinician meetings, all staff trainings, trainings where all 250 employees of our clinic gather together quarterly and we had our CEO give us updates and trainings about what to do,” said participant No. 325 located in the Northeast.
**Theme 4: physician empowerment**	
Engaging in multilevel advocacy	“I talked to a lot of patients that are not actually immigrants about protecting one another and asking them if they would also like to have the rapid response network phone number in their phone to help protect the community,” said participant No. 318 located in the West.
	“There’s a resource from [organization names] that I had a hand in editing…I forward [the resource] to folks,” said participant No. 341 located in the West.
	“There are residents from different specialties who have come together to try and mobilize and plan how to form a first responder coalition, not wearing our hats as medical providers but as members of the community…,” said participant No. 330 located in the West.
Leveraging trust	“What’s been more surprising is that people feel comfortable telling me they’re scared because saying that in this world is a political statement. It means so much that they feel comfortable sharing that with me…,” said participant No. 321 located in the Northeast.
	“…my patients have been with our institution for a long time, so they really trust us and are used to coming to see us, so I don’t get met with resistance when I refer a patient to a specialist,” said participant No. 362 located in the South.
Role modeling for trainees and peers	“I did organize this large talk for both of our main campuses about…ethical issues facing undocumented patients,” said participant No. 362 located in the South.
	“Getting better at training our colleagues is something I’m really passionate about and making sure that we can exercise our rights as physicians and protecting HIPAA,” said participant No. 341 located in the West.
Protecting patient privacy	“As soon as November [2024] came around, I said, ‘We aren’t ready [and] I need more support; our patients need more support.’ My clinic manager…whipped out this laminated page [designating private spaces] and stuck it everywhere…,” said participant No. 341 located in the West.
	“Now nothing is safe anymore. We don’t know what’s going to happen with all the [medical] records…use caution and don’t put unnecessary information in the [medical records],” said participant No. 322 located in the Northeast.

Abbreviations: ACLU, American Civil Liberties Union; CEO, chief executive officer; DHHS, US Department of Health and Human Services; HIPAA, Health Insurance Portability and Accountability Act; ICE, US Immigration and Customs Enforcement; TPS, temporary protected status.

**Figure.**
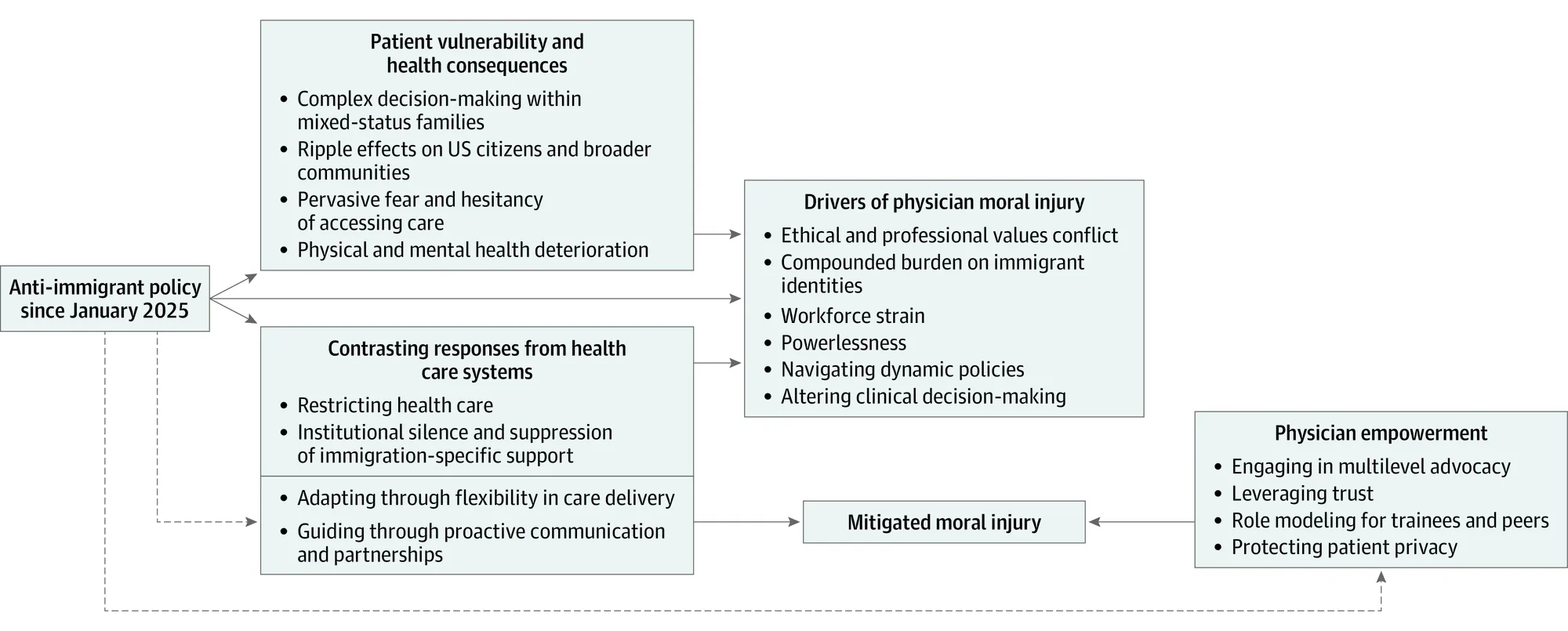
Conceptual Model Displaying the Drivers and Mitigators of Physician Moral Injury Due to Anti-Immigrant Policies Anti-immigrant policies drove patient vulnerability and health consequences and unsupportive health care system responses, leading to physician moral injury. In contrast, some health care systems adapted through supportive responses, which mitigated moral injury. Moral injury was also mitigated by physicians' responses to anti-immigrant policies.

**Table 3.** Participant Recommendations for Physicians, Health Care Systems, and Policymakers to Improve Immigrant Care

Domain	Participant recommendations
**Recommendations for physicians**	
Documentation	Do not document immigration status in the EHR Limit documentation of non–clinically relevant information in the EHR Disable artificial intelligence transcription during sensitive history taking and encounters
Care delivery	Offer telehealth services and minimize in-person visits Consolidate appointments, laboratory assessments, and medication access (eg, mail order prescriptions or prescription delivery) Partner with community-based organizations (eg, immigrant rights, housing) to provide supportive services Offer vetted resources to patients (eg, Know Your Rights Card^34^)
Communication	Use trauma-informed communication strategies Provide medical necessity letters when appropriate
Advocacy	Engage in multilevel advocacy through professional organizations, coalitions, and committees Center lived experience in advocacy efforts Train students, residents, and peers on immigrant health and advocacy Develop and distribute immigrant health best practice tool kits
**Recommendations for health care systems**	
Documentation	Share and implement best practice policies to limit sensitive documentation Reinforce data protection and privacy protocols
Care delivery	Expand telehealth and flexible scheduling options Deliver community-based care Offer vetted resources to patients Strengthen interdisciplinary and community partnerships
Communication	Communicate institutional policies Provide immigrant-affirming messaging and supportive signage Provide structured opportunities to process moral injury (eg, debriefing sessions, peer support)
Capacity building	Diversify funding resources Hire staff with language and cultural concordance
**Recommendations for policymakers**	
Documentation	Prohibit data sharing between health care systems and ICE Strengthen legal protections for health data confidentiality
Care delivery	Invest in social needs infrastructure (eg, food insecurity, job training, housing, education) Expand federal and state insurance eligibility and universal health care initiatives Enact permanent policies that cover flexible care (eg, telehealth) Reinstate the protected areas policy Ensure comprehensive health care to ICE detainees Enforce judges’ decisions about asylum cases
Communication	Include health care constituents, health care workers, and people with lived experience in policy development, policy briefs, and legislative testimonies Convey the restrictions and limitations regarding new legislation

Abbreviations: EHR, electronic health record; ICE, US Immigration and Customs Enforcement.

### Theme 1: Drivers of Physician Moral Injury

#### Ethical and Professional Values Conflict

Participants described a tension between immigrant- and health-related messaging and policies, the care they could provide, and their commitment to human dignity. Participant No. 352 located in the Northeast said, “We go into family medicine because we believe that everyone deserves to be healthy regardless of anything about them…and to be told that is not allowed, and see people face the consequences of living under a government that doesn’t see them as equal and deserving to be healthy is very frustrating.” This sentiment resonated for participant No. 319 located in the West who said, “I work at a public hospital. I represent the government. The government is not doing things that are beneficial for people right now…. We post signs that say ‘Care is here. Care is for everybody.’ But when the rubber hits the road there’s a real risk that ICE may show up in our lobby….”

#### Compounded Burden on Immigrant Identities

Participants with immigrant identities reported feeling additional distress because they also felt targeted by policies. When describing working while there were ongoing ICE raids nearby, participant No. 317 located in the West stated, “It put more stress [on me], because I am also an immigrant…. I’m going to care for [immigrants], but I’m in the line of danger.” Participant No. 340 located in the West reported, “I was born in the US but I’m Middle Eastern…. I look brown. I’m not going to do things that I normally do alone because I don’t want to get picked up. My kids rely on me.”

#### Workforce Strain

Participants reported feeling emotional and administrative strain. They reported being deeply affected by how anti-immigrant policies led to staff quitting, layoffs, and distress. Participant No. 356 located in the Northeast described, “We have had some [staff] who left…they couldn’t stand the heartbreak.” Participants reported being strained to meet the needs of patients and mitigate policy harms. Participant No. 341 located in the West described sacrificing personal time to provide comprehensive care, “Last night I charted until 12:30 am because I decided to use my charting time to spend with patients…. I won’t always do that, but right now a little bit more love is warranted, a little bit more care, a little bit more attention, a little bit more trust building.” Participant No. 333 located in the Northeast wanted to maximize visits in case patients could not access future care, “It makes the job harder, and it’s already a hard job, because we have 20-minute visits but I also feel like I don’t know when this person will interact with health care again [so I try to do everything in 1 visit].”

#### Powerlessness

Participants reported feeling powerless caring for immigrant patients. Participant No. 358 located in the South shared, “the work my office used to do has been weaponized and demonized in the local news.” Participant No. 362 located in the South said, “There’s a heaviness to all of it. I’m talking about these things that are so much bigger than me, and not within my area of expertise.” Participant No. 335 located in the West discussed that the lack of agency made her rethink her job, stating “…outside of [clinic] I have no control…it’s made me question continuing if it changes that drastically [and] I can’t take care of everyone that walks in the door.”

#### Navigating Dynamic Policies

Participants described challenges navigating evolving federal policy proposals, with health systems being confused about legality or implementing anticipated policies before they took effect. Participant No. 334 located in the West stated, “We’re not lawyers, we’re not legal experts…and the lawyers don’t even know what to say right now…[we are] trying to keep up with what they’re throwing at us.” Participant No. 333 located in the Northeast shared their frustration with shifting privacy standards (referring to the Centers for Medicare & Medicaid Services sharing data with DHS), “We had always communicated to patients that their information was private…and that’s clearly not the case.”

#### Altering Clinical Decision-Making

Participants shared how their clinical decision-making was affected by considering immigration-related policies. Participant No. 343 located in the West described, “Would I rather have them be at home while they’re having substernal, crushing chest pain vs what are the costs and benefits of going [to the hospital]?” Participant No. 340 located in the West shared, “The loss of access to insurance and treatments is impacting my ability to provide care.” Although participants attempted to maintain best practice standards, participant No. 325 located in the Northeast reported having to alter care to reduce patient risk, stating “I had to switch injection medications to pill form [to] minimize them coming to clinic.”

### Theme 2: Patient Vulnerability and Health Consequences

#### Complex Decision-Making Within Mixed-Status Families

Participants reported on the difficulty patients in mixed-status families experienced when making decisions. Participant No. 322 located in the Northeast recalled a patient crying when asked why she had missed appointments, “If she doesn’t work, she won’t get paid…they’re going to lose their apartment, and her adult son and daughter-in-law are both in the process of being deported…what’s going to happen to the grandkids?” Participant No. 339 located in the West described, “the person who has citizenship goes to work or to the grocery store,” but the family remains fearful because US citizens are also being detained by ICE.

#### Ripple Effects on US Citizens and Broader Communities

Participants commented on the community-level financial and health effects beyond immigrant patients. Participant No. 334 located in the West described a White, nonimmigrant, male patient and small business owner whose employees were missing work out of deportation fear despite many being US citizens, noting how the patient had stopped taking his medications and that “…the immigration stress [was] bleeding out into the entire community.” Participant No. 352 located in the Northeast described, “no one really feels safe if they could be perceived as someone who could be here illegally….”

#### Pervasive Fear and Hesitancy of Accessing Care

Participants reported patients missing and canceling appointments; preferring telehealth; avoiding urgent care; not answering clinic calls; and declining referrals, laboratory work, and pharmacy visits due to fear. Participant No. 324 located in the Northeast shared, “People are asking for virtual visits, hesitant to be sent to other places when they require a referral, and hesitant to go to the emergency department.” Participant No. 367 located in the West noted the fear extended to losing future care, “She was worried that she wasn’t going to be able to get the surgery…. We talked about how we could move [the surgery] up.”

#### Physical and Mental Health Deterioration

Participants described worsening health outcomes among patients with chronic conditions. Participant No. 325 located in the Northeast said, “There are physical health impacts [including] poorly controlled diabetes and worsening blood pressure control due to not getting checkups.” Participants also reported a surge in mental health diagnoses and exacerbations reflected by increases in “PHQ-9 [9-Item Patient Health Questionnaire] and GAD-7 [7-Item General Anxiety Disorder] scores,” as noted by participant No. 362 located in the South, “trouble sleeping,” as noted by participant No. 326 located in the Northeast, “panic attacks,” as noted by participant No. 319 located in the West, and “more suicidality,” as noted by participant No. 356 located in the Northeast.

### Theme 3: Contrasting Responses From Health Care Systems

#### Restricting Health Care

Participants described health care systems restricting immigrants’ access to care. When describing the effects of threatened funding cuts to clinics that care for undocumented patients, participant No. 356 located in the Northeast shared, “[federally qualified health centers] have shut down doing this work entirely out of fear.” The restrictions included telehealth noted participant No. 351 located in the West, “Because of the political climate, we are not allowed to provide telehealth anymore….”

#### Institutional Silence and Suppression of Immigration-Specific Support

Participants noted that health care systems remained silent on immigration-related topics (eg, policies about ICE presence, resources to support immigrant patients) due to risk aversion. Participant No. 342 located in the West said, “One of the downsides of us being dependent on federal funding is that [leadership is] very cautious about doing anything that is counter to federal policies…. No one wanted to risk anything.” Other health systems actively discouraged immigration-specific support, including visible signage and resources. Participant No. 322 located in the Northeast said, “We have been told we can’t hand out those red cards—the Know Your Rights Card^34^ [a card explaining individuals’ rights if they are stopped by immigration or police]—because that would be seen as obstructing government, so all we can do is direct people to the patient resource center.”

Health systems also prevented action that made participants feel censored. Participant No. 357 located in the Northeast said, “they issued a directive…not to attempt to assist patients who are being sought by federal immigration authorities.” The culture of institutional silence and inaction further contributed to physicians being fearful about retribution for their public efforts such as supporting immigrant causes via signing petitions or participating in marches. Participant No. 345 located in the West said, “I’m trying to be strategic about what I sign or post on Facebook because I want to do this work as long as possible and I am worried that…the government could find out and that could be dangerous.”

#### Adapting Through Flexibility in Care Delivery

In contrast, other participants highlighted how health care systems facilitated access. Participant No. 317 located in the West shared, “My staff has been great at switching [patients] to telehealth.” This participant also reported that staff mailed resources, referrals, and medications to patient homes. Participant No. 356 located in the Northeast described an innovative care model that is “…building hubs in community centers for telehealth so that people don’t need their own [technology].” Clinics also developed recommendations about medical record documentation. Participant No. 342 located in the West reported, “[our clinic is] vocal and open about not obtaining specific [immigration] information or making documentation tied to identities.”

#### Guiding Through Proactive Communication and Partnerships

Participants identified helpful health care system guidance including proactive communication. Participant No. 340 located in the West said, “We’ve been kept well informed about what things would impact our ability to care for patients….” Participant No. 367 located in the West noted the importance of partnerships with community-based organizations who “are already doing the work of finding out what people need in the community.”

### Theme 4: Physician Empowerment

#### Engaging in Multilevel Advocacy

Participants reported engaging in advocacy efforts through clinical work, networks, partnerships, and political engagement. For example, participant No. 327 located in the Northeast “wrote a letter of medical necessity [for a patient detained by ICE] which said that without medications [the patient was] at risk for another stroke” and the patient was released from ICE detention. Participant No. 356 located in the Northeast created a family preparedness plan and disseminated it, indicating “It is available in our charting system, and translated into different languages.”

Participants reported that engaging in broader efforts outside of clinical work also provided agency. Participant No. 367 located in the West said, “I testified in Congress on [ICE not coming to hospitals] and then also wrote this op-ed about it.” Participant No. 351 located in the West described taking on multiple regional and national roles with various organizations to “be the voice of my patients.” Participant No. 342 located in the West mobilized with her colleagues “to open a clinic that’s not dependent on federal funding [given restrictions on health systems that accept federal funds]….”

#### Leveraging Trust

Participants were motivated by the trust that patients and the public have in physicians and felt compelled to act. Participant No. 347 located in the Northeast shared, “You’re going to have to be much more proactive and preventive with people and realize that you’re a voice of authority that people trust in and [consider] how we use that for good?”

#### Role Modeling for Trainees and Peers

Participants also engaged in educational efforts. Participant No. 321 located in the Northeast shared, “My focus has been on trying to support the residents…how to advocate and helping them become really strong physicians who support the care of our community.” Participant No. 352 located in the Northeast said, “We’re trying to figure out how to teach doctors to be better community organizers and better partners with communities if we really want anything to change and keep things from getting worse.”

#### Protecting Patient Privacy

Participants commented on adaptations they have made to protect patient care and privacy. Participant No. 352 located in the Northeast noted the importance of “keeping [clinic] doors closed so that [the clinic] isn’t an open space [for ICE].” Participant No. 362 located in the South described, “there will be times when I pause [the artificial intelligence scribe] if they’re talking about specific details…. I try not to document documentation status and not record any details related to immigration or travel plans.”

## Discussion

In this study, physicians described how anti-immigrant policies since January 2025 have generated profound moral injury stemming from ethical tensions, workforce strain, and a sense of powerlessness. These policies shaped institutional responses that contributed to moral injury, with some health systems restricting care, remaining silent, or discouraging support for undocumented patients. Witnessing the detrimental effects of these policies on the health of immigrants, mixed-status families, and US citizens also exacerbated physician moral injury. Physicians also identified mitigating pathways, including advocacy and supportive health system responses.

Moral injury arises when clinicians are unable to provide care aligned with their ethical values—a tension acutely amplified by anti-immigrant policy.^26^ Moral injury has been documented amid systemic constraints, including resource limitations and institutional barriers.^24,25,35,36,37^ Our study extends these findings by identifying anti-immigrant policy as a distinct and underexamined structural driver of moral injury. Increasing moral injury among physicians, who already face elevated risk for burnout, is concerning because it affects workforce retention, care delivery, and patient outcomes.^38,39,40,41,42^ Participants with immigrant identities, who are underrepresented in the health care workforce, expressed moral injury particularly strongly, reflecting their dual identities as clinicians and members of the targeted community, and warrant further attention and institutional support.^43,44^ Nonphysician staff, who represent a diverse workforce with less institutional power and heightened exposure to anti-immigrant policies, may also bear disproportionate moral injury that warrants further investigation.^45^

Our study demonstrates how moral injury can be mitigated and agency restored through individual- and system-level adaptations. Physicians who engaged in advocacy, collective organizing, and student and peer education described gaining agency, suggesting that action-oriented responses can combat the feelings of powerlessness that contributed to moral injury.^46,47,48,49^ This dynamic was mirrored at the systems level. Health care systems that responded proactively through transparent communication, flexibility, privacy protections, and proimmigrant messaging mitigated moral injury, supported retention of immigrant and mixed-status family patients, and reinforced organizational trust, which were findings consistent with prior work.^50,51^ Conversely, health care systems that remained silent or implemented anticipated federal anti-immigrant policies, intensified moral injury, eroded trust, and contributed to staff turnover and fear.^45,52,53,54,55^ Our findings position moral injury not only as a consequence of anti-immigrant policy, but as a modifiable condition responsive to individual action and institutional support. Participants’ recommendations for physicians, health care systems, and policymakers to improve immigrant care span documentation, care delivery, communication, advocacy, and capacity building and offer a practical framework of actionable strategies that can mitigate moral injury and reduce harm to patients and the workforce caring for them (Table 3).

Addressing modifiable drivers of moral injury stemming from anti-immigrant policies through individual- and systems-level responses can strengthen access to care and workforce well-being. However, responsibility cannot rest primarily on individual physicians. Health system leaders and policymakers have the opportunity and obligation to respond to anti-immigrant policies in ways that protect patients, sustain the workforce, and uphold the integrity of health care institutions.

### Limitations

This study has several limitations. First, we recruited participants through email using purposive and snowball sampling, which may have led to selection bias with participants who were motivated or aligned with the study being more likely to participate. In addition, this recruitment strategy may have resulted in only participants from 13 states and largely from urban, academic settings in the West and Northeast being represented.

Second, we focused on primary care physicians; perspectives from other specialists and staff may vary. Third, we asked physicians to describe the experiences of immigrant patients, resulting in secondhand accounts that may not capture the lived experiences of patients.

## Conclusions

In this qualitative study, physicians experienced moral injury stemming from anti-immigrant policies starting in January 2025, which was compounded for those with immigrant identities and because of health care systems’ varying responses to these policies. Physicians perceived that changes in immigration policy have adversely affected immigrant and US citizens. Despite these challenges, physicians and health care systems have adapted to support patients and staff, which has mitigated moral injury.

## Notes

Supplement 1.
Participant interview guide

Supplement 2.
Data sharing statement
