# Supplementary material for: Moral Injury Among Physicians Caring for Immigrant Patients Amid Anti-Immigrant Policies
Source: JAMA Intern Med. 2026 Jul 20:e262899. Online ahead of print. doi: 10.1001/jamainternmed.2026.2899 (PMC13386305; doi:10.1001/jamainternmed.2026.2899)
Supplement: Supplement 1. — Participant interview guide [file jamainternmed-e262899-s001.pdf]

Supplemental Online Content

Martín M, Ambriz L, Gonzalez Ramirez M, et al. Moral injury among physicians caring for immigrant patients amid anti-immigrant policies. Published online July 20, 2026. *JAMA Intern Med*. doi:10.1001/jamainternmed.2026.2899

Participant Interview Guide

This supplemental material has been provided by the authors to give readers additional information about their work.

© 2026 Martín M et al. *JAMA Internal Medicine*.

Participant Interview Guide

1. What are you seeing in clinic because of federal and state policies related to immigration since January 2025?
2. What mental or physical health changes are you seeing in patients, if any?
3. What do patients bring up?
4. What changes, if any, have you noticed in patients' willingness to seek medical care?
5. Have changing immigration policies impacted the care you deliver?
6. How, if at all, has your clinic adapted to changing immigration policies?
7. Have these changes impacted you?
8. Have you taken any actions in response?
9. Do you have recommendations to improve the care of immigrant patients in the current climate?

© 2026 Martín M et al. *JAMA Internal Medicine*.
