# Supplementary material for: Moral Injury Among Physicians Caring for Immigrant Patients Amid Anti-Immigrant Policies
Source: JAMA Intern Med. 2026 Jul 20:e262899. Online ahead of print. doi: 10.1001/jamainternmed.2026.2899 (PMC13386305; doi:10.1001/jamainternmed.2026.2899)
Supplement: Supplement 2. — Data sharing statement [file jamainternmed-e262899-s002.pdf]

# Data Sharing Statement

Martín. Moral Injury Among Physicians Caring for Immigrant Patients Amid Anti-Immigrant Policies. *JAMA Intern Med*. Published July 20, 2026. doi:10.1001/jamainternmed.2026.2899

**Data**
**Data available:** No

**Additional Information**
**Explanation for why data not available:** The data are qualitative interview transcripts.
